# Soybean oil, a linoleic acid source, in lamb diets: metabolic, feeding behavior, and ruminal parameters

**DOI:** 10.5194/aab-68-77-2025

**Published:** 2025-02-06

**Authors:** Victor Guimarães Oliveira Lima, Liliane Oliveira da Silva, José Esler de Freitas Jr., Henry Daniel Ruiz Alba, Vagner Maximino Leite, Willian Pereira Silva, Douglas dos Santos Pina, Laudí Cunha Leite, Carlindo Santos Rodrigues, Stefanie Alvarenga Santos, Gleidson Giordano Pinto de Carvalho

**Affiliations:** 1 Department of Animal Science, Universidade Federal da Bahia, Salvador, Bahia, 40170-110, Brazil; 2 Department of Animal Science, Universidade Federal do Recôncavo da Bahia, Cruz das Almas, Bahia, 44380-000, Brazil

## Abstract

​​​​​​​​​​​​​​The objective of this study was to evaluate the effects of including soybean oil (SO) in the diet of feedlot lambs on metabolic, behavioral, and ruminal parameters. Five rumen-cannulated lambs (average body weight of 47.76 ± 7.14 kg at approximately 15 months of age) were distributed in a 5 × 5 Latin square design. Their diets consisted of increasing levels of SO inclusion: 0, 30, 60, 90, and 120 g kg^−1^ of dry matter (DM). Each period lasted 21 d, with 14 d for adaptation and 7 d for data collection. The data were analyzed using linear and quadratic polynomial contrasts, except for the ruminal fermentation parameters and plasma glucose, which were analyzed using repeated measures over time. There was a linear reduction in DM ( P = 0.019) and crude protein ( P = 0.007) intake as well as a linear decrease in non-fibrous carbohydrate intake ( P < 0.001) and digestibility ( P = 0.039) due to increased SO inclusion levels. The intake of ether extract increased linearly ( P < 0.001), while its digestibility showed a quadratic relationship ( P < 0.001) with the inclusion of SO in the diet. Feeding behavior was not altered by SO inclusion, except for rumination efficiency, which decreased linearly ( P < 0.001). There was a linear reduction in nitrogen intake ( P = 0.007), while no influence ( P > 0.05) of SO inclusion was observed on ruminal fermentation parameters, except for a linear decrease in the concentration of rumen ammonia nitrogen ( P = 0.023). The use of SO in diets for feedlot lambs should be approached with caution, as it can reduce DM intake, which may lead to decreased animal performance; however, further studies are needed to determine the effects on the productive cost and performance.

## 1 Introduction

Brazil has been the largest producer of soybeans in the world in recent years, and production is expected to grow at a rate of 1.2 % a^−1^ over the next decade (OECD/FAO, 2021). Soybeans undergo various methods (mechanical and/or chemical) to extract oil (18 %–20 % extractable oil), which has a favorable fatty acid (FA) profile (Chuffa et al., 2014), with linoleic acid being the most abundant FA, comprising approximately 52.91–59.1 g per 100 g of the total FA content (Oliveira et al., 2021; Lima et al., 2024).

In recent decades, there has been an increase in the number of studies evaluating the inclusion of vegetable oils in diets for ruminants (Bionaz et al., 2020). The importance of including lipids in diets lies in their ability to increase the energetic density of the diet and enhance energy, as evidenced by reductions in methane production and increases in the propionate concentration (Goulas et al., 2001; Vargas et al., 2020; Bahramkhani-Zaringoli et al., 2022). At the ruminal level, the inclusion of soybean oil (approximately 7 % ether extract) in the diets of growing lambs has been found to promote greater development of papillary width (33.6 % greater), epithelial thickness (57.1 % greater), submucosal layer thickness (60.9 % greater), and muscle layer thickness (50.1 % greater), compared with a diet without soybean oil inclusion (Bahramkhani-Zaringoli et al., 2022).

However, the results obtained from including soybean oil in ruminant diets are controversial. In vitro studies have shown favorable effects of 60 g of soybean oil per kilogram of dry matter (DM) on ruminal fermentation, as a trend to reduce the rumen ammonia nitrogen concentration (Gómez-Cortés et al., 2008), while in vivo studies have indicated greater ruminal energy efficiency due to a reduced acetate : propionate ratio (Barletta et al., 2016; Bettero et al., 2017; Felix-Bernal et al., 2016; Sharifi et al., 2018). Furthermore, soybean oil inclusion has been found to reduce methane production (Lima et al., 2019) and to have no affect on DM intake or fiber digestibility (Ferreira et al., 2016; Freitas et al., 2018; Dias et al., 2020).

Recent studies have indicated a reduction in voluntary DM intake and in the digestibility of nutritional compounds due to soybean oil addition (Van Cleef et al., 2016; Parente et al., 2020), which can change the ruminal metabolism (Zhao et al., 2016). Rumen bacteria use the biohydrogenation (BH) mechanism as their primary method to mitigate the negative effects of polyunsaturated fatty acids (PUFAs) in the rumen (with linoleic acid biohydrogenation rates of around 89 %), producing vaccenic acid (18 : 1 *trans*-11), which can partially inhibit BH and increase PUFA flux to the intestine (Ferreira et al., 2016; Zhao et al., 2016; Freitas et al., 2018). In the liver, the metabolizable energy produced from PUFAs is utilized by animals to achieve maximum productive efficiency (Allen, 2020). It is important to note that the adequate transition and adaptation of animals to an oil-based diet can facilitate the proper adaptation of rumen microbes and the physiological functions of the rumen epithelium (Bahramkhani-Zaringoli et al., 2022).

In this context, we hypothesize that feedlot lambs can be fed higher levels of soybean oil in their diets without it negatively affecting their performance; moreover, it may even potentially promote better performance due to increased metabolic energy, as we believe that lambs can tolerate higher levels of dietary oil. Therefore, the present study was conducted to evaluate the effects of the including soybean oil, a source of linoleic acid, on the metabolic, feeding behavior, and ruminal parameters of feedlot lambs.

## 2 Material and methods

### 2.1 Location and ethical considerations

The study was conducted at the Experimental Farm of the Federal University of Bahia (UFBA), located in São Gonçalo dos Campos, Bahia, Brazil (12°23^′^57.51^′′^ S, 38°52^′^44.66^′′^ W). All experimental procedures involving animals were approved by the Ethics Committee on Animal Use of the School of Veterinary Medicine and Animal Science at UFBA (approval no. 72/2018).

### 2.2 Animals, experimental diets, and design

Five male Santa Inês lambs with cannulas (2 in. latex cannula with an external diameter of 15 cm, an internal diameter of 13 cm, and a 7.5 cm usable opening; Kehl^®^, São Carlos, São Paulo, Brazil) were used in this study (average age of 15 months; average initial body weight of 47.76 ± 7.14 kg – mean ± standard deviation). The animals were housed in covered pens (1.2 m^2^, suspended with slatted wooden floors) equipped with drinkers and feeders, with ad libitum access to water and feed. Upon arrival, the lambs were identified; immunized with a polyvalent vaccine against *Clostridium*; subjected to parasite control (endoparasites and ectoparasites); and supplemented with vitamins A, D, and E. The lambs were assigned to the diets according to a 5 × 5 Latin square design.

**Table 1:** Centesimal composition of ingredients and chemical composition of experimental diets.

Item	Soybean oil level (g kg^−1^ DM)				
	0	330	660	1000	120
Composition (g kg^−1^ DM)					
Sorghum silage	400	400	400	400	400
Soybean meal	116	120	124	128	133
Ground corn	461	427	393	359	324
Soybean oil	0	30	60	90	120
Urea	8	8	8	8	8
Mineral mixture^a^	15	15	15	15	15
Chemical composition (g kg^−1^ DM)					
Dry matter (g kg^−1^ on an as-fed basis)	663.3	667.8	672.4	677.0	681.7
Mineral matter	41.6	41.5	41.3	41.1	41.0
Crude protein	148.9	147.9	146.8	145.7	145.0
Ether extract	30.9	59.6	88.2	116.9	145.5
Neutral detergent fiber ap^b^	273.9	270.7	267.5	264.3	261.1
Acid detergent fiber ap^b^	131.2	130.7	130.1	129.6	129.0
Hemicellulose	142.7	140.0	137.4	134.7	132.0
Cellulose	111.3	110.9	110.5	110.1	109.7
Lignin	19.9	19.8	19.6	19.5	19.4
Indigestible neutral detergent fiber	75.3	75.0	74.6	74.3	73.9
Non-fibrous carbohydrates	519.1	494.9	470.7	446.6	421.9
Total digestible nutrients	754.6	774.6	794.6	814.6	834.5
Metabolizable energy (Mcal kg^−1^)	3.0	3.2	3.4	3.5	3.7
Neutral detergent insoluble protein (g kg^−1^ CP)	80.0	78.8	77.5	76.2	74.8
Acid detergent insoluble protein (g kg^−1^ CP)	18.6	18.4	18.3	18.1	17.8
Fatty acid profile (grams per 100 g of total FAs)					
Caprylic (C8 : 0)	0.01	0.01	0.01	0.01	0.01
Capric (C10 : 0)	0.05	0.05	0.05	0.04	0.04
Lauric (C12 : 0)	0.08	0.08	0.07	0.07	0.07
Miristic (C14 : 0)	0.56	0.55	0.54	0.53	0.52
Palmitic (C16 : 0)	4.67	4.88	5.09	5.29	5.50
Palmitoleic (C16 : 1)	0.12	0.12	0.12	0.12	0.13
Estearic (C18 : 0)	1.15	1.24	1.34	1.43	1.53
Oleic (C18 : 1 n-9 )	7.95	8.17	8.40	8.62	8.83
Linoleic (C18 : 2 n-6 )	12.72	13.81	14.89	15.98	17.06
α -Linolenic (C18 : 3 n-3 )	0.72	0.91	1.10	1.29	1.48

^a^ Guaranteed mineral levels (per kilogram of active elements) are as follows: calcium – 110 g, phosphorus – 87 g, sulfur – 18 g, copper – 590 mg, cobalt – 15 mg, sodium – 147 g, chromium – 20 mg, iodine – 50 mg, manganese – 2 g, selenium – 20 mg, zinc – 3.8 g, fluorine (max) – 870 mg, and molybdenum – 300 mg. ^b^ “Neutral detergent fiber ap” and “Acid detergent fiber ap” denote the respective neutral and acid detergent fiber expressed exclusive of ash and protein content.

The study comprised five 21 d periods, totaling 105 d. Each 21 d period consisted of 14 d for the adaptation of the lambs to the diets (adaptation period) and 7 d for data collection (experimental period). The diets included five levels of soybean oil: 0, 30, 60, 90, and 120 g kg^−1^ DM (Table 1). The diets were formulated to be isonitrogenous and to meet the lambs' requirements for an average daily gain (ADG) of 200 g d^−1^ (NRC, 2007).

Diets were composed of sorghum silage (*Sorghum bicolor* (L.) Moench) as roughage and soybean meal, ground corn, soybean oil, urea, and a specific mineral supplement for sheep as components of the concentrate. Diets were mixed daily to prevent soybean oil rancidity and were supplied twice a day in equal portions (at 08:00 and 16:00 LT, UTC - 3). The initial body weight (IBW) of the animals was recorded on the first day of each experimental period after a 16 h fast.

### 2.3 Sampling collection and chemical analyses

Samples of supplied diets and refusals were collected weekly in each experimental period. The samples were pre-dried in a forced-air-circulation oven at 55 °C for 72 h. Air-dried samples were ground in a Wiley mill using a 1 mm sieve. Chemical analysis were conducted for DM (test method 934.01), ether extract (EE; test method 920.39), crude protein (CP; test method 981.10), mineral matter (test method 930.05), and lignin (method-973.18), following the methods described by the Association of Official Analytical Chemists (AOAC, 2002).

Neutral detergent fiber (NDF) and acid detergent fiber (ADF) were determined using heat-stable amylase (for NDF only) and expressed exclusive of residual ash and protein (NDFap and ADFap, respectively) according to the methodologies of Mertens (2002) and Licitra et al. (1996). Hemicellulose and cellulose were estimated from the values obtained from NDF, ADF, and lignin. Non-fibrous carbohydrates (NFCs) and total digestible nutrients (TDNs) were estimated using the equation proposed by Hall (2000) and Cruz et al. (2021), respectively. Digestible and metabolizable energy were estimated from TDN values (NRC, 2001).

Ingredient samples were freeze-dried and uniformly ground (for 10–15 s using a grinder – Cadence 150W MDR 302) to perform the fatty acid profile analysis for the diets, following the methodologies and equipment described in Alba et al. (2021) and Nascimento et al. (2021).

### 2.4 Intake and apparent digestibility of nutrients

The dry matter intake was calculated from the weights of the supplied feed and the refusals. Nutrient intake was determined using the following formula: intake (kg) = nutrient_ingested_ - nutrient_refusal_. The apparent digestibility trial was conducted between days 17 and 19 of each experimental period, utilizing the total feces collection method.

Fecal collection bags were employed to gather the total feces excreted during the trial. The bags were opened twice daily (at 07:00 and 14:00 LT) to remove and store the feces in a freezer at - 20 °C until further analysis. Prior to analysis, fecal samples were pre-dried in a forced-air oven at 55 °C for 72 h. Samples were then ground in a Wiley knife mill (model 0.48, Marconi, Piracicaba, Brazil) using a 1 mm screen and stored for subsequent analysis. To estimate the apparent digestibility of the nutritional compounds, the following formula was used: [(nutrient intake - nutrient in feces) / nutrient intake] × 100.

### 2.5 Feeding behavior

Feeding behavior was evaluated on day 15 of each experimental period. Individual observers conducted 24 h observations at 5 min intervals to determine the time spent on rumination, feeding, and idling (where the latter denotes the time spent not performing the other two activities). Eight trained observers (divided into groups of two, rotating every 3 h) were positioned to minimize disturbance to the lambs' behavior. The environment was artificially lit prior to data collection to help the animals adapt.

For these assessments, the ruminal boluses of each animal were monitored during three different periods of the day (09:00–11:00, 16:00–18:00, and 20:00–22:00 LT), and the time spent on each ruminated cud was recorded. Feeding efficiency, rumination efficiency, total daily chewing time (h d^−1^), the number of ruminated chews per day, and the number of ruminating chews per day were calculated, as proposed by Polli et al. (1996) and Bürger et al. (2000).

### 2.6 Urine sampling and nitrogen balance

The total collection method was employed to obtain urine from the lambs on day 16 of each experimental period. For this method, funnels and conductive hoses were attached to collect urine in plastic containers containing 100 mL of 20 % H_2_SO_4_ (Plaizier et al., 2000). The urine from each animal was weighed, homogenized, filtered through two layers of cheesecloth, and stored at - 20 °C for further analysis.

The nitrogen content in the urine and fecal samples was determined following method 948.13, as proposed by AOAC (1990). The nitrogen balance was calculated by considering the nitrogen intake and the nitrogen in urine and feces.

### 2.7 Ruminal fermentation and blood parameters

On day 20 of each experimental period, ruminal samples were obtained from different regions of the rumen (cranial sac, ventral sac, and dorsal sac). Rumen collections were performed every 2 h, starting with the morning feeding (point 0) and continuing for 12 h. The rumen content was filtered through four layers of cheesecloth (1 mm) to obtain the ruminal fluid. Immediately after collection, the pH was measured using a digital pH meter (ORP 8651, AZ Instrument Corp., Tanzi District, Taichung, Taiwan).

Ruminal fluid samples were centrifuged (16 000 × g for 15 min at 4 °C; 80-2B centrifuge, Centribio, São Paulo, São Paulo, Brazil), and the supernatant was stored for further analysis. Volatile fatty acid (VFA) concentrations were measured using a high-performance liquid chromatograph (SPD-10A VP, Shimadzu, Tokyo, Japan) equipped with an ultraviolet detector (wavelength 210 nm) and a Supelco C18 column (30 cm × 7.9 mm, 0.8 mL min^−1^ flow rate, 117 kgf, kilogram force, pressure). Sulfuric acid (1 %) in water was used as the mobile phase (Mathew et al., 1997). Concentrations of ruminal ammonia nitrogen were assessed using a colorimetric method, according to Foldager (1977). To estimate methane (CH ${}_{4})$ gas production, the following equation was used: CH_4_ = 0.45 (acetate) - 0.275 (propionate) + 0.40 (butyrate) (Moss et al., 2000).

On day 21 of each experimental period, blood samples (10 mL) were collected from the jugular vein into sterile Vacutainer tubes without anticoagulant (BD Vacutainer^®^ SST II Advance) for serum samples and into tubes with a glycolytic inhibitor (BD Vacutainer^®^ Fluoreto) for plasma samples. Blood was centrifuged (80-2B centrifuge, Centribio, São Paulo, São Paulo, Brazil) at 3500 × g for 15 min at room temperature to obtain plasma samples.

Serum urea and plasma glucose concentrations were determined using colorimetric methods with commercial kits from Labtest (Ref. 104 and 133, respectively; Labtest Diagnóstica S.A., Minas Gerais, Brazil) and a spectrophotometer. Urea in urine samples was determined following the same method, with specific modifications for urine samples.

### 2.8 Statistical analysis

A 5 × 5 Latin square design was utilized, referring to the five diets with soybean oil inclusion (0, 30, 60, 90, and 120 g kg^−1^ DM) across five periods. The results were analyzed using the PROC MIXED command in the Statistical Analysis System (SAS) software (version 9.4). The effects of soybean oil inclusion in the diet of feedlot lambs were analyzed using linear and quadratic polynomial contrasts, adopting 0.05 as the critical level of probability. The data were analyzed using the following model:

1 $$Y_{ijk}=\mu +\beta_{i}+P_{j}+\alpha_{k}+\varepsilon_{ijk},$$

where $Y_{ijk}$ is the observed value of the dependent variable, μ is the overall mean, $\beta_{i}$ is the random effect of animal i ( i=1 to 5), $P_{j}$ is the random effect of period j ( j=1 to 5), $\alpha_{k}$ is the fixed effect of treatment k ( k=1 to 5 diets), and $\varepsilon_{ijk}$ is the residual error.

Rumen ammonia nitrogen, VFA, pH, and plasma glucose data were analyzed as repeated measures over time (0, 2, 4, 6, 8, 10, and 12 h relative to the morning feeding) using the MIXED procedure in SAS, with the following model:

2 $$Y_{ijk}=\mu +\beta_{i}+P_{j}+\alpha_{k}+T_{l}+T_{l}\times \alpha_{k}+\varepsilon_{ijkl},$$

where $Y_{ijk}$ is the observed value of the dependent variable, μ is the overall mean, $\beta_{i}$ is the random effect of animal i ( i=1 to 5), $P_{j}$ is the random effect of period j ( j=1 to 5), $\alpha_{k}$ is the fixed effect of treatment k ( k=1 to 5 diets), $T_{l}$ is the fixed effect of time l ( l=0 , 2, 4, 6, 8, 10, and 12 h after feeding), $T_{l}\times \alpha_{k}$ is the effect of the time and treatment interaction, and $\varepsilon_{ijkl}$ is the residual error. For all evaluations, a 0.05 probability was considered for the type-I error.

## 3 Results

### 3.1 Intake and apparent digestibility of nutrients

There was a linear reduction in the intake of DM ( P = 0.015), CP ( P = 0.005), NDFap ( P = 0.006), and NFCs ( P < 0.001) as a function of soybean oil inclusion levels. The EE intake ( P < 0.001) increased by 1.178 g for every 1 g kg^−1^ DM of soybean oil included in the diet. However, soybean oil inclusion did not influence TDN intake ( P > 0.05). Conversely, for every 1 g kg^−1^ DM of soybean oil included in the diet, there was a reduction in DM intake ( P = 0.039) of 0.0030 g kg^−1^ of body weight (BW) and a reduction in NDF intake ( P = 0.011) of 0.0011 g kg^−1^ BW (Table 2).

**Table 2:** Intake and digestibility of nutritional compounds in feedlot lambs fed diets containing increasing levels of soybean oil. SEM denotes the standard error of the mean.

Intake (kg)	Soybean oil level (g kg^−1^ DM)					SEM	P value	
	0	30	60	90	120		Linear	Quadratic
Intake (g d^−1^)								
Dry matter^a^	1286.7	1390.6	1272.7	1164.6	1140.9	57.493	0.015	0.276
Crude protein^b^	199.9	212.3	196.0	171.0	169.3	9.483	0.005	0.318
Ether extract^c^	42.5	90.4	125.9	155.1	186.9	10.514	< 0.001	0.365
Neutral detergent fiber^d^	317.3	338.5	291.5	264.8	260.3	17.728	0.006	0.648
Non-fibrous carbohydrates^e^	672.7	691.4	606.2	526.2	477.8	23.526	< 0.001	0.357
Total digestible nutrients	967.9	1159.3	1069.4	1060.9	1072.6	56.512	0.546	0.213
Intake (g kg^−1^ body weight)								
Dry matter^f^	2.4	2.6	2.4	2.3	2.2	0.121	0.039	0.386
Neutral detergent fiber^g^	0.6	0.6	0.5	0.5	0.5	0.034	0.011	0.735
Digestibility coefficient (%)								
Dry matter	75.1	78.5	74.9	79.2	76.4	2.548	0.704	0.641
Crude protein	72.8	78.2	76.1	79.7	79.1	2.166	0.061	0.451
Ether extract^h^	83.8	91.6	93.8	95.5	94.2	1.147	< 0.001	0.001
Neutral detergent fiber	50.1	54.7	43.9	57.1	52.1	5.130	0.701	0.805
Non-fibrous carbohydrates^i^	88.4	90.1	87.2	86.7	84.2	1.540	0.034	0.326
Total digestible nutrients^j^	76.2	83.5	84.2	92.6	93.5	2.566	< 0.001	0.609

The regression equations used are as follows: ^a^ DM_intake_ = 1354.64 - 1.7255 × SO, $R^{2}$ = 0.77; ^b^ CP_intake_ = 210.23 - 0.3418 × SO, $R^{2}$ = 0.82; ^c^ EE_intake_ = 49.44 + 1.1781 × SO, $R^{2}$ = 0.99; ^d^ NDF_intake_ = 332.02 - 0.6256 × SO, $R^{2}$ = 0.84; ^e^ NFCs_intake_ = 705.88 - 1.8501 × SO, $R^{2}$ = 0.95; ^f^ DMintake_g kg^−1^ BW_: $\hat{Y}$ = 2.5632 - 0.00296 × SO, $R^{2}$ = 0.68; ^g^ NDF_g kg^−1^ BW_: $\hat{Y}$ = 0.6256 - 0.00108 × SO, $R^{2}$ = 0.77; ^h^ EE_digestibility_: $\hat{Y}$ = 84.1659 + 0.26 × SO - 0.00148SO^2^, $R^{2}$ = 0.72; ^i^ NFCs_digestibility_: $\hat{Y}$ = 89.6532 - 0.03886 × SO, $R^{2}$ = 0.74; ^j^ TDNs: $\hat{Y}$ = 77.2276 + 0.1457 × SO, $R^{2}$ = 0.93.

The apparent digestibility of DM, CP, and NDFap was not significantly affected ( P > 0.05) by increasing levels of soybean oil in the animals' diet. The apparent digestibility of EE showed a quadratic effect ( P = 0.001), and TDNs ( P < 0.001) increased linearly with soybean oil inclusion in the lambs' diet. EE digestibility increased with the inclusion of up to 87.5 g kg^−1^ DM of soybean oil, reaching a maximum digestibility of 95.6 %. Beyond this inclusion level, EE digestibility began to decrease. The apparent digestibility of NFCs was reduced ( P = 0.034) by 0.0390 % for every 1 g kg^−1^ DM of soybean oil included in the diet. Total digestible nutrients linearly increased ( P < 0.001) by 0.146 % for every 1 g of soybean oil added to the diet (Table 2).

### 3.2 Feeding behavior

No significant changes ( P > 0.05) were detected in feeding, ruminating, or idling times with soybean oil inclusion in the lambs' diet. There was also no effect ( P > 0.05) on DM feeding efficiency. However, the rumination efficiency of DM ( P = 0.006) and rumination efficiency of NDF ( P < 0.001) were linearly reduced with soybean oil inclusion, by 0.377 g DM h^−1^ and 0.1195 g NDF h^−1^ for every 1 g of soybean oil added to the diet, respectively. The number of mericic chews and chewing time were not influenced ( P > 0.05) by soybean inclusion in the animals' diet (Table 3).

**Table 3:** Feeding behavior of feedlot lambs fed diets containing increasing levels of soybean oil. SEM denotes the standard error of the mean.

Item	Soybean oil level (g kg^−1^ DM)					SEM	P value	
	0	30	60	90	120		Linear	Quadratic
Time (h d^−1^)								
Feeding	3.1	2.5	2.3	2.8	2.9	0.366	0.970	0.151
Rumination	6.2	7.9	7.5	6.7	7.4	0.447	0.444	0.180
Idling	14.7	13.6	14.2	14.5	13.7	0.653	0.609	0.909
Efficiency (g DM h^−1^)								
Feeding	465.3	611.5	584.3	419.9	415.2	60.683	0.154	0.077
Rumination^a^	208.5	179.5	169.3	176.4	153.5	10.564	0.006	0.477
Efficiency (g NDF h^−1^)								
Feeding	114.4	149.3	132.6	94.8	95.2	15.794	0.087	0.153
Rumination^b^	51.2	43.5	38.6	40.1	35.0	2.365	< 0.001	0.213
Chewing								
Grams of DM per cud	2.3	1.9	2.0	1.9	2.0	0.144	0.154	0.271
Number of chews per cud	60.0	61.7	66.4	59.7	68.8	3.256	0.191	0.810
Mericic chew time per cud	40.2	39.6	43.7	40.7	47.2	2.546	0.124	0.507
Number of ruminated cuds	567.4	732.1	628.6	605.5	577.3	47.701	0.492	0.113
Total chewing time (h d^−1^)	9.3	10.4	9.8	9.5	10.3	0.653	0.609	0.909

The regression equations used are as follows: ^a^ RE_g DM h^−1^_: $\hat{Y}$ = 200.06 - 0.377 × SO, $R^{2}$ = 0.81; ^b^ RE_g NDF h^−1^_: $\hat{Y}$ = 48.8432 - 0.1195 × SO, $R^{2}$ = 0.85.

### 3.3 Nitrogen balance and blood metabolite concentrations

Nitrogen intake ( P = 0.005) and fecal nitrogen excretion ( P = 0.010) were linearly reduced by soybean oil inclusion in the diet. The amounts of absorbed nitrogen, urinary nitrogen, retained nitrogen, and urinary urea were not significantly affected ( P > 0.05) by soybean oil inclusion in the diet of feedlot lambs. The average nitrogen retention was 13.1 g d^−1^ (Table 4).

**Table 4:** Nitrogen balance and blood metabolites of feedlot lambs fed diets containing increasing levels of soybean oil. SEM denotes the standard error of the mean.

Item	Soybean oil level (g kg^−1^ DM)					SEM	P value	
	0	30	60	90	120		Linear	Quadratic
Nitrogen intake (g d^−1^)^a^	32.0	34.0	31.4	27.4	27.1	1.517	0.005	0.318
Fecal nitrogen (g d^−1^)^b^	8.9	7.4	7.6	5.9	5.4	0.872	0.010	0.965
Absorbed nitrogen (g d^−1^)^c^	23.1	26.6	23.8	21.5	21.7	1.337	0.085	0.250
Urinary nitrogen (g d^−1^)	10.4	10.6	10.6	9.5	10.0	1.107	0.625	0.915
Retained nitrogen (g d^−1^)^d^	12.7	16.0	13.2	11.9	11.7	1.685	0.294	0.412
Urinary urea (mg dL^−1^)	15.4	14.9	16.6	14.2	16.0	1.835	0.937	0.950
Blood metabolites								
Serum urea (mg dL^−1^)	42.5	46.1	47.3	44.2	46.0	3.480	0.650	0.554
Plasma glucose (g dL^−1^)	80.3	75.4	78.1	78.4	74.2	2.879	0.322	0.920

The regression equations used are as follows: ^a^ nitrogen intake: $\hat{Y}$ = 33.6356 - 0.05467 × SO, $R^{2}$ = 0.87; ^b^ fecal nitrogen: $\hat{Y}$ = 8.7124 - 0.02823 × SO, $R^{2}$ = 0.91.^c^ Absorbed nitrogen = nitrogen intake - fecal nitrogen. ^d^ Retained nitrogen = urinary nitrogen - absorbed nitrogen.

Blood metabolic parameters were not significantly influenced ( P > 0.05) by increasing soybean oil inclusion in the lambs' diet, with average values of 45.2 mg dL^−1^ for serum urea and 77.3 mg dL^−1^ for plasma glucose, respectively (Table 4).

A time effect was observed for plasma glucose ( P < 0.001) with soybean oil inclusion (Fig. 3).

### 3.4 Ruminal fermentation

There was no significant effect among the soybean inclusion levels on the following ruminal fermentation parameters: pH ( P = 0.196), VFAs ( P = 0.706), acetate ( P = 0.706), propionate ( P = 0.721), butyrate ( P = 0.100), the acetate : propionate ratio ( P = 0.872), and methane ( P = 0.241). The plasma glucose ( P = 0.411) content was also not influenced by soybean oil inclusion. However, the ruminal ammonia nitrogen concentrations ( P = 0.023) decreased linearly by 0.02 mg dL^−1^ % for every 1 g of soybean oil added to the diet (Table 5).

**Table 5:** Ruminal parameters of feedlot lambs fed diets containing increasing levels of soybean oil. SEM denotes standard error of the mean.

Item	Soybean oil level (g kg^−1^ DM)					SEM	P value				
	0	30	60	90	120		Diet	Time	D × T	Linear	Quadratic
pH	6.0	6.1	6.3	6.2	6.1	0.110	0.196	< 0.001	0.860	0.316	0.058
Rumen ammonia nitrogen (mg dL^−1^)^a^	11.0	11.3	8.8	8.7	9.4	1.300	0.023	< 0.001	0.504	0.011	0.194
Volatile fatty acids (mM)	62.2	64.4	60.8	62.0	61.4	2.620	0.706	0.762	0.998	0.485	0.900
Acetate (moles per 100 mol of VFAs)	74.2	74.3	74.4	74.1	74.4	1.720	0.936	0.254	0.893	0.759	0.959
Propionate (moles per 100 mol of VFAs)	15.7	15.0	15.3	15.0	15.3	1.031	0.721	0.191	0.663	0.508	0.347
Butyrate (moles per 100 mol of VFAs)	10.1	10.7	10.1	10.9	10.3	0.457	0.100	0.229	0.950	0.280	0.238
Acetate : propionate ratio	5.1	5.3	5.1	5.3	5.1	0.400	0.872	0.244	0.303	0.795	0.532
Methane (% of VFAs)^b^	21.0	21.7	20.4	21.8	20.6	0.760	0.241	0.584	0.954	0.675	0.628

The regression equation used was as follows: ^a^ rumen ammonia nitrogen: $\hat{Y}$ = 10.9960 - 0.0195 × SO, $R^{2}$ = 0.55. ^b^ Methane was estimated according to Moss et al. (2000): CH_4_ = 0.45(acetate) - 0.275(propionate) + 0.40(butyrate).

**Figure 1:**
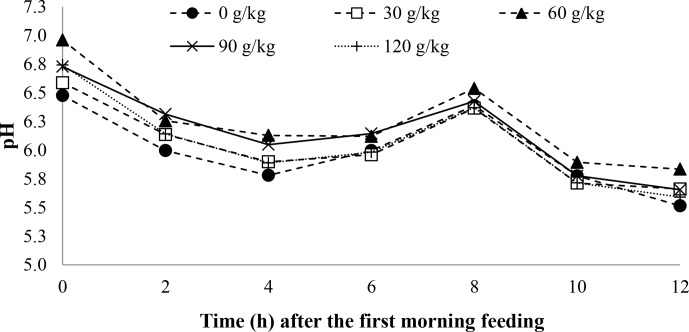
The effect of time on the rumen pH of feedlot lambs fed diets containing increasing levels of soybean oil. Here, SEM = 0.110, P value_diet_ = 0.196, P value_time_ < 0.001, and P value_diet×time_ = 0.860.

**Figure 2:**
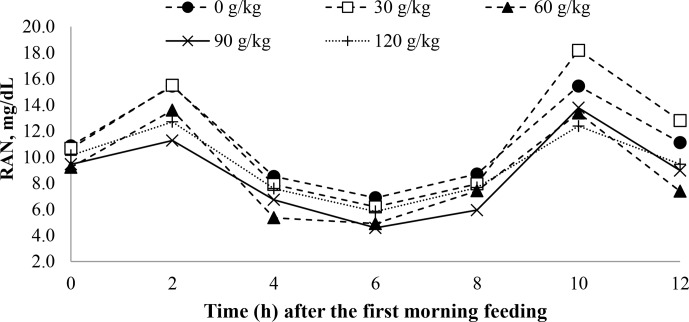
The effect of time on the rumen ammonia nitrogen (RAN) of feedlot lambs fed diets containing increasing levels of soybean oil (SO). Here, SEM = 1.300, P value_diet_ = 0.023, P value_time_ < 0.001, and P value_diet×time_ = 0.504.

A time effect was observed for pH ( P < 0.001; Table 5) and ruminal ammonia nitrogen concentrations ( P < 0.001; Table 5) with soybean oil inclusion (Figs. 1 and 2). The pH decreased as a function of time, reaching its lowest point at 4 h after feeding. In contrast, the ruminal ammonia nitrogen concentrations increased 2 h after feeding, followed by a subsequent decrease. No significant time effect was observed for the VFA concentration ( P = 0.762), acetate ( P = 0.254), propionate ( P = 0.191), butyrate ( P = 0.229), the acetate : propionate ratio ( P = 0.244), or methane ( P = 0.584).

**Figure 3:**
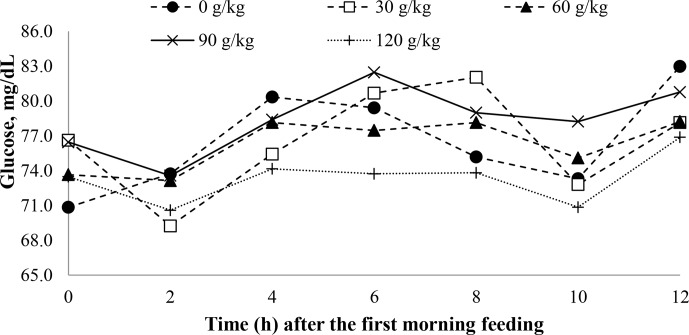
The effect of time on the plasma glucose concentrations of feedlot lambs fed diets containing increasing levels of soybean oil (SO). Here, SEM = 2.630; P value_diet_ = 0.411, P value_time_ < 0.001, and P value_diet×time_ = 0.166.

## 4 Discussion

### 4.1 Intake and apparent digestibility of nutrients

The DM intake reduction with the inclusion of soybean oil in the animals' diet can be attributed to the increased supply of long-chain fatty acids (LCFAs), as evidenced by the higher intake of EE. This increase in EE provides an energy source for the metabolism of lambs. This behavior can be explained using the hepatic oxidation theory, which suggests that the increase in energy from oxidizing acetyl coenzyme A from the addition of soybean oil and the increase in LCFAs increase adenosine triphosphate (ATP) production, leading to hypophagic effects (Allen, 2014). PUFA-rich diets increase cholecystokinin (CCK) peptide hormone release, which promotes decreased gastric emptying and increased rumen retention time to improve nutrient digestibility. However, a negative effect is observed due to rumen distension and satiety receptor activation promoted by increased retention time (Allen et al., 2009). As no alterations in the apparent digestibility of NDF were observed, it is possible that the nutrient content in the diet did not play a role in regulating DM intake through the physical mechanism of ruminal distension.

It is important to highlight that the inclusion of 41.4 g kg^−1^ DM soybean oil in the animals' diet promotes an average daily gain of 200 g d^−1^, whereas the inclusion of 59.17 g kg^−1^ DM results in average daily gains of 180 g d^−1^ (Lima et al., 2024). In the present study, using a regression equation, the inclusion levels of 41.4 and 59.17 g kg^−1^ DM correspond to calculated DM intakes of 1283.2 and 1252.5 g d^−1^, respectively. Thus, while the inclusion of soybean in the diet of feedlot lambs leads to a decrease in DM intake, the inclusion of 59.17 g kg^−1^ DM will support the necessary DM intake to achieve an average daily gain of 180 g d^−1^.

The increase in the apparent digestibility of EE and TDNs may be directly linked to the enhanced flow of LCFAs to the small intestine (Ferreira et al., 2016; Freitas et al., 2018). According to Alba et al. (2021), LCFAs exhibit high digestibility in the small intestine.

The fibrolytic microbiota of the rumen can be negatively impacted by the presence of unsaturated fatty acids in an animal's diet. However, linoleic acid, the main unsaturated fatty acid found in soybean oil, has a high biohydrogenation rate (89 % to 98 %) in the rumen (Barletta et al., 2016; Bettero et al., 2017; Freitas et al., 2018). Therefore, it can be concluded that the fibrolytic microbiota was not adversely affected by the inclusion of soybean oil in the animals' diet and, as a result, the digestibility of NDF remained unchanged. Similar findings in the literature indicate that sources of linoleic acid have less detrimental effects on the fibrolytic microbiota compared with other fatty acid sources, such as linolenic acid (Ferreira et al., 2016; Zhao et al., 2016).

The reduction in NFC digestibility associated with soybean inclusion is related to a decreased supply of fermentative substrates. The inclusion of soybean oil lowers the starch and amino acid content of the diet, leading to a reduction in crude protein intake. The greater degradability of NFCs occurs at the ruminal level and is negatively affected by the decreased intake of amino acids, creating unfavorable conditions for the growth of NFC-fermenting microbiota (Andreazzi et al., 2018).

### 4.2 Feeding behavior

The reduction in consumption suggests a strong indication of increased satiety with the inclusion of soybean oil in the diet of lambs. However, this was not reflected in feeding, rumination, or idle times. Despite the unchanged durations of these behaviors, feeding and rumination efficiencies for NDF were reduced, indicating that, although the time spent feeding and ruminating remained constant, the effectiveness of these activities was lower.

The maintenance of the DM content per rumination bolus, alongside the similar number of chews, time spent on mericic chews per rumination bolus, and the reduction in fecal N excretion, points to a physiological adaptation that enhances nutrient availability by increasing the exposure area of DM particles consumed by the lambs. This aligns with findings from Lima et al. (2019), who observed similar patterns in feedlot lambs consuming diets with 50 g of soybean oil kg^−1^ DM.

### 4.3 Nitrogen balance and blood metabolite concentrations

The reduction in nitrogen intake can be attributed to the decreased DM intake, given that the diets were isonitrogenous. The fact that absorbed nitrogen and retained nitrogen were not affected by soybean oil inclusion suggests an enhancement in the efficiency of nitrogen utilization, leading to potential economic and environmental benefits (Newbold and Ramos-Morales, 2020).

The reduction in nitrogen intake likely prompted physiological adaptations that improved nitrogen utilization efficiency in small ruminants (Van Soest, 1994; Da Silva et al., 2020). This is reflected in the consistent nitrogen uptake across treatments, explaining the observed nitrogen balance despite reduced intake.

Although nitrogen intake decreased linearly, this effect was not physiologically significant in promoting differences in serum urea between treatments. Regarding plasma glucose, propionic acid concentrations were similar between treatments, resulting in comparable plasma glucose levels across the treatments.

### 4.4 Ruminal fermentation

Although the inclusion of soybean oil reduced the consumption of NFCs, there was no corresponding increase in the ruminal pH. This likely due to the maintenance of the VFA concentration, which is supported by the sustained rumination time (min d^−1^), total chewing time, and number of ruminated cuds. The inclusion of linoleic acid sources, such as soybean oil, generally does not significantly affect the ruminal pH, primarily because LCFAs are not utilized as fermentative substrates for the VFA concentration (Zhao et al., 2016; Parvar et al., 2017; Sharifi et al., 2018).

The processes of the isomerization and the biohydrogenation of PUFAs in soybean oil (Ferreira et al., 2016; Zhao et al., 2016; Freitas et al., 2018) were likely not efficient enough to modulate ruminal fermentation significantly. Consequently, concentrations of VFAs, acetate, propionate, butyrate, the acetate : propionate ratio, and methane remained stable despite the inclusion of soybean oil.

Moreover, the maintenance of gluconeogenic compounds, such as propionate, contributed to stable blood glucose levels in the lambs. However, as soybean oil levels increased in the animals' diet, nitrogen compounds showed a reduction in nitrogenous ammonia concentrations, likely due to decreased crude protein intake. This reduction indicates a lesser contribution of fermentative substrates for ruminal ammonia-nitrogen-producing microorganisms, supporting the findings of Ferreira et al. (2016), who linked lower ruminal ammonia nitrogen concentrations to reduced nitrogen excretion, given that increased excretion often correlates with higher rumen ammonia nitrogen levels (Rufino et al., 2020).

## 5 Conclusion

The inclusion of soybean oil, a source of energy and linoleic acid, in diets consisting of 60 % concentrate and 40 % roughage for feedlot lambs should be approached with caution, as it can reduce the animals' dry matter intake and is not effective at modulating ruminal fermentation, which may lead to lower animal performance. However, to promote average daily gains of 200 g d^−1^, a maximum inclusion of 41.4 g kg^−1^ DM of soybean oil is recommended. Furthermore, additional studies are necessary to determine the optimal use of soybean oil in lamb diets.

## Data Availability

The original data used in this study are available from the corresponding author upon request.
